# Stimulation of peripheral Kappa opioid receptors inhibits inflammatory hyperalgesia via activation of the PI3Kγ/AKT/nNOS/NO signaling pathway

**DOI:** 10.1186/1744-8069-8-10

**Published:** 2012-02-08

**Authors:** Thiago M Cunha, Guilherme R Souza, Andressa C Domingues, Eleonora U Carreira, Celina M Lotufo, Mani I Funez, Waldiceu A Verri, Fernando Q Cunha, Sergio H Ferreira

**Affiliations:** 1 Department of Pharmacology, School of Medicine of Ribeirão Preto University of São Paulo, Av. Bandeirantes, 3900, 14049-900, Ribeirão Preto SP, Brazil; 2 Universidade Federal de Uberlândia, Instituto de Ciências Biomédicas, Rua Pará 1720, Bloco 2A, piso superior, Umuarama 38405-320, Uberlândia, MG - Brazil; 3 School of Ceilandia, University of Brasilia, QNN 14, Área Especial, 72220-140, Ceilândia Sul Ceilândia - DF, Brazil; 4 Departamento de Ciências Patológicas, Centro de Ciências Biológicas, Universidade Estadual de Londrina, Rod. Celso Garcia Cid PR 445, Km 380 Cx., Postal 6001, 86051-990 Londrina, Parana, Brazil

## Abstract

**Background:**

In addition to their central effects, opioids cause peripheral analgesia. There is evidence showing that peripheral activation of kappa opioid receptors (KORs) inhibits inflammatory pain. Moreover, peripheral μ-opioid receptor (MOR) activation are able to direct block PGE_2_-induced ongoing hyperalgesia However, this effect was not tested for KOR selective activation. In the present study, the effect of the peripheral activation of KORs on PGE_2_-induced ongoing hyperalgesia was investigated. The mechanisms involved were also evaluated.

**Results:**

Local (paw) administration of U50488 (a selective KOR agonist) directly blocked, PGE_2_-induced mechanical hyperalgesia in both rats and mice. This effect was reversed by treating animals with L-NMMA or N-propyl-L-arginine (a selective inhibitor of neuronal nitric oxide synthase, nNOS), suggesting involvement of the nNOS/NO pathway. U50488 peripheral effect was also dependent on stimulation of PI3Kγ/AKT because inhibitors of these kinases also reduced peripheral antinociception induced by U50488. Furthermore, U50488 lost its peripheral analgesic effect in PI3Kγ null mice. Observations made in vivo were confirmed after incubation of dorsal root ganglion cultured neurons with U50488 produced an increase in the activation of AKT as evaluated by western blot analyses of its phosphorylated form. Finally, immunofluorescence of DRG neurons revealed that KOR-expressing neurons also express PI3Kγ (≅ 43%).

**Conclusions:**

The present study indicates that activation of peripheral KORs directly blocks inflammatory hyperalgesia through stimulation of the nNOS/NO signaling pathway which is probably stimulated by PI3Kγ/AKT signaling. This study extends a previously study of our group suggesting that PI3Kγ/AKT/nNOS/NO is an important analgesic pathway in primary nociceptive neurons.

## Background

Inflammatory pain is primarily due to the sensitization of specific classes of nociceptive neurons by the direct action of inflammatory mediators (e.g., prostaglandins). In this context, pharmacologic control of inflammatory pain in the periphery is mainly based on two principal strategies. First, the use of non-steroidal anti-inflammatory drugs (aspirin and aspirin-like drugs) inhibits cyclooxygenase-derived prostaglandin production and, consequently, reduces nociceptor sensitization [1]. This effect ultimately prevents the development of hyperalgesia (decrease in nociceptive threshold) in humans and animals. On the other hand, the second strategy is exemplified by some analgesic drugs, like opioids and dipyrone, which are able to directly block ongoing nociceptor sensitization through peripheral actions [2,3]. In fact, local (intraplantar, i.pl.) administration of opioids reversed already established hyperalgesia induced by prostaglandin E_2 _(PGE_2_) [2,4]. Therefore, in contrast to aspirin-like drugs that act through the prevention of nociceptor sensitization by inhibiting prostaglandin synthesis, opioids are able to directly block ongoing inflammatory hyperalgesia.

In an attempt to elucidate this mechanism, we showed that the inhibition of neuronal nitric oxide synthase inhibits peripheral antinociception achieved with opioids, suggesting the participation of nitric oxide [4,5]. These pharmacological data are further supported by the observation that the peripheral analgesic effect of morphine is lost in nitric oxide deficient mice [4]. Further addressing the molecular basis of opioid peripheral analgesia, we recently demonstrated that the phosphoinositide 3-kinase gamma (PI3Kγ)/AKT signaling pathway is the first step between the activation of μ-opioid receptors by morphine and selective agonists and the stimulation of nitric oxide control of peripheral analgesia.

There are evidences in the literature showing that activation of kappa opioid receptors (KORs) also inhibits inflammatory pain [6,7]. For instance, selective KOR agonist reduces carrageenin-induced hyperalgesia in rats [6]. Furthermore, the mechanism operating this effect seems to be dependent on nitric oxide synthase (NOS)/nitric oxide (NO) signaling pathway [6]. However, it is not clear which NOS isoform is involved in this effect and if PI3Kγ/AKT signaling is involved. Therefore, in the present study we tested if the peripheral activation of KORs also directly blocks ongoing inflammatory hyperalgesia induced by PGE_2_. The molecular mechanisms involved in this effect were also investigated.

## Methods

### Animals

The experiments were performed in male Wistar rats (180-200 g), C57BL/6 wild type (WT) male mice (20-25 g) and PI3Kγ deficient mice (PI3Kγ-/-). All animals were housed in the animal care facility of the Faculty of Medicine of Ribeirão Preto-University of Sao Paulo. The animals were taken to the testing room at least 1 h before the experiments and were used only once. Food and water were available ad libitum. The animal care and handling procedures were in accordance with the International Association for the Study of Pain guidelines [8] for those animals used in pain research, which were approved by the Committee for Ethics in Animal Research of the Faculty of Medicine of Ribeirão Preto-USP.

### Nociceptive test

#### The electronic pressure-meter test

The mechanical nociceptive threshold was tested in mice and rats as previously reported [9,10]. In a quiet room, mice or rats were placed in acrylic cages (12 × 10 × 17 cm) with wire grid floors 15-30 min before the start of testing. The test consisted of evoking a hindpaw flexion reflex with a hand-held force transducer (electronic aesthesiometer; IITC Life Science, Woodland Hills, CA) adapted with a 0.5 (mice) or 0.7 mm^2 ^(rats) polypropylene tip. The investigator was trained to apply the tip perpendicularly to the central area of the hindpaw with a gradual increase in pressure. The endpoint was considered removal of the paw followed by clear flinching movements. After paw withdrawal, the intensity of the pressure was automatically recorded, and the final value for the response was obtained by averaging three measurements. The animals were tested before and after treatments. The results are expressed as the delta (Δ) withdrawal threshold (in g), calculated by subtracting the zero-time mean measurements from the time interval mean measurements. The withdrawal threshold was 9.1 ± 0.2 g (mice; mean ± SEM.; n = 30) and 38.8 ± 0.7 (rats; mean ± SEM.; n = 20) before injection of the hypernociceptive agents. Quantification of nociception was performed by a blinded observer. Multiple paw treatments with saline did not alter the basal reaction time, which was similar to that observed in non-injected paws.

#### Thermal nociceptive test

The latency of paw withdrawal to radiant heat stimuli was measured using a Plantar Ugo Basile apparatus (Stoelting, Wood Dale, Il) as previously described [11]. Mice can move freely in this apparatus on an elevated glass surface with plastic boxes above as the top cover. Mice are given a one h acclimation period prior to testing until they become calm and motionless. A calibrated infrared light source of high intensity was applied perpendicular on the plantar surface of each mouse's hind paw. The end point was characterized by the removal of the paw followed by clear flinching movements. Latency to paw withdrawal was automatically recorded. Each hind paw was tested alternately with an interval of 5 min for four trials. Paw withdrawal latency of four trials from both hind paws of each mouse was averaged and recorded as mean ± SEM.

#### Primary DRG neuron culture

Rats weighing 100 g were euthanized by decapitation under isoflurane anesthesia. Dorsal root ganglia were collected (18-20 ganglia per animal) and transferred to a sterile Hank's balanced salt solution (Sigma, St Louis, MO, EUA) containing HEPES 10 mM (HBSS/HEPES). Isolated ganglia were incubated with collagenase III (0.28 U/mL) for 75 min and trypsin (0.25% w/v) for 12 min in HBSS/HEPES. Ganglia were washed and resuspended in Dulbecco's Modified Eagle medium (DMEM, Sigma, USA) containing 10% fetal bovine serum and penicillin/streptomycin 1000 U/mL. Cells were dissociated by triturating the ganglia with a fire polished pipette and then were plated in six well plastic plates coated with Matrigel (BD, USA). Cell cultures were maintained at 37°C and 5% CO_2_, and experiments were performed within 24 h [12].

#### Western blot analysis

After the indicated stimulation, DRG cells were homogenized in a lysis buffer containing a mixture of protease inhibitors and phosphatase inhibitors (Sigma). The protein concentrations of the lysate were determined using a BCA Protein Assay kit (Pierce, Rockford, IL), and 30 μg of protein was loaded for each lane. Protein samples were separated on a SDS-PAGE gel (10% gradient gel; Bio-Rad, Hercules, CA) and transferred to nitrocellulose membranes (Amersham Pharmacia Biotech, Little Chalfont, UK). The filters were blocked with 5% dry milk and incubated overnight at 4°C with a primary antibody, phosphorylated (p-Ser473) AKT (1:500; Cell Signaling Technology, Beverly, MA), for 1 h at room temperature (RT) with an HRP-conjugated secondary antibody (1:20000; Jackson ImmunoResearch, PA, USA). The blots were visualized in an ECL solution (Amersham Pharmacia Biotech) for 2 min and exposed to film (Hyperfilm, Amersham Pharmacia Biotech) for 1-30 min. A nonphosphorylated AKT (1:1000; Cell Signaling Technology, Beverly, MA) antibody was used as a loading control [13].

#### DRG immunohistochemistry

Animals were terminally anesthetized with urethane and perfused through the ascending aorta with saline, followed by 4% paraformaldehyde in a 0.1 M phosphate buffer, pH 7.4 (4°C). After the perfusion, DRGs were removed and postfixed in the same fixative for 2 h before being replaced overnight with 20% sucrose. All of the DRGs were embedded in OCT, and DRG sections (14 μm) were cut in a cryostat and processed for immunofluorescence. All of the sections were blocked with 2% BSA in 0.3% Triton X-100 for 1 h at room temperature and incubated for 2 h at 4°C with a mixture of polyclonal rabbit anti-PI3Kγ (1:200; Santa Cruz, CA, USA), polyclonal goat anti-Kappa opioid receptors (1:200; Santa Cruz), antibodies, and, finally, a mixture of AlexaFluo-488 and AlexaFluo-594 conjugated secondary antibodies (Molecular Probes, Carlsbad, CA, USA) for 1 h at room temperature.

#### Drugs

The following drugs were obtained from the sources indicated. Wortmannin, L-NMMA, naloxone chlorhydrate, LY294002, Complete Freund's Adjuvant (CFA), Nor-Binaltorphimine (Nor-BNI) and PGE_2 _were purchased from Sigma Chemical Co. (St. Louis, MO, USA). AS605240 and AKT inhibitor IV were obtained from Calbiochem (San Diego, CA, USA). *N*^ω^-Propyl-L-arginine was obtained from Tocris Bioscience (Ellisville, MO, USA).

#### Data analyses and statistics

All results are presented as the means ± S.E.M. The experiments were repeated at least twice. A two-way analysis of variance (ANOVA) was used to compare the groups and doses at all times curves when the hypernociceptive responses were measured at different times after the stimulus injection. The factors analyzed were treatments, time and time by treatment interaction. When there was a significant time by treatment interaction, a one-way ANOVA followed by a Bonferroni's *t*-test was performed at each time. Alternatively, when the hypernociceptive responses were measured once after the stimulus injection, the differences between responses were evaluated by a one-way ANOVA followed by a Bonferroni's *t*-test. *P *< 0.05 was considered as significant.

## Results

### Activation of peripheral KORs inhibits hyperalgesia induced by PGE_2 _in rats and mice

**Figure 1:**
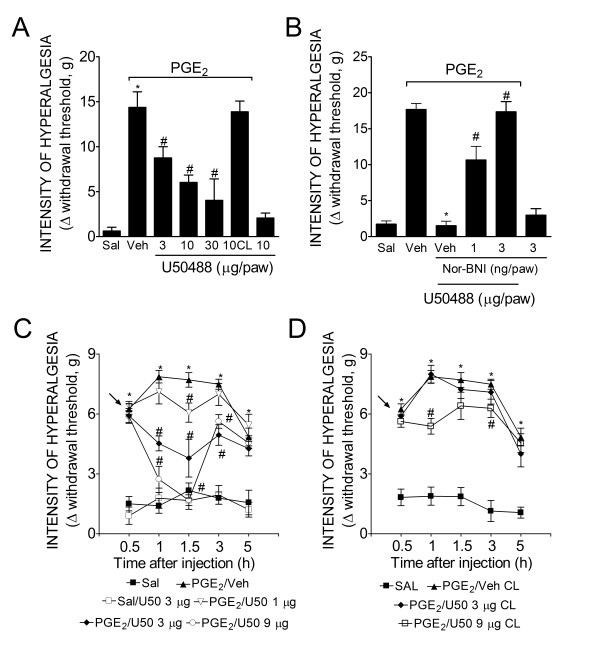
**KOR agonist (U50488) directly blocked PGE_2_-induced inflammatory hyperalgesia in rats and mice**. (**A**) Mechanical hyperalgesia in rats was induced by an ipl. injection of PGE_2 _(100 ng/paw)_. _After 2 h, U50488 (3-30 μg/paw) was administrated into the ipsilateral or contralateral (CL) paws. Mechanical hyperalgesia was evaluated 1 h after U50488 injection using the electronic von Frey test. (**B**) The antinociceptive effect of a local injection of U50488 (10 μg/paw, 2 h after PGE_2 _injection) upon PGE_2_-induced hyperalgesia was prevented by treatment with a selective antagonist of KOR (Nor-BNI; 30 min before U50488 injection) **(C-D) **Mechanical hyperalgesia was induced by an ipl. injection of PGE_2 _(30 ng/paw) in mice. After 30 min, U50488 (1-9 μg/paw) was administrated into the ipsilateral or contralateral (CL) paws. Data are expressed as the mean ± S.E.M. of 5-6 animals per group. * Indicates statistical significance compared to the saline group. # Indicates statistical significance compared to the PGE_2 _control group. *P *< 0.05, one-way ANOVA, followed by the Bonferroni correction.

In the first part of this study, we tested the effect of U50488 (a selective agonist of KORs) on the established inflammatory hyperalgesia produced by PGE_2 _in rats and mice. We previously demonstrated that opioids post-treatment (2 h after the PGE_2 _paw injection in rats and 30 min after the injection in mice) are effective in reducing mechanical hyperalgesia produced by PGE_2 _[4]. The local administration of U50488 inhibited PGE_2_-induced mechanical hyperalgesia in a dose-dependent manner in rats (Figure 1A) and in mice (Figure 1C) (3-30 μg/paw and 1-9 μg/paw, respectively).The dose of U50488 that produced antinociception after PGE_2_-induced hyperalgesia when injected in the ipsilateral paw of rats was ineffective when injected in the contralateral paw, suggesting a peripheral site of action (Figure 1A). The peripheral antinociceptive effect of U50488 was reversed by local administration of a selective antagonist of KOR, Nor-BNI (Figure 1B). This local effect was also observed in mice at doses of 3 and 9 μg of U50488; however, the dose of 9 μg was able to produce systemic effects (Figure 1D).

### nNOS/NO pathway mediates Antinociception produced by the peripheral activation of KORs

**Figure 2:**
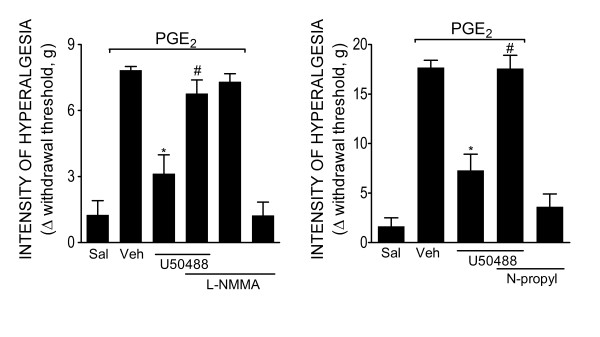
**The nNOS/nitric oxide pathway participates in the peripheral antinociceptive effect of a KOR agonist**. (**A**) Mechanical hyperalgesia in mice was induced by an ipl. injection of PGE_2 _(30 ng/paw). The antinociceptive effect of a local injection of U50488 (3 μg/paw, 30 min after PGE_2 _injection) after PGE_2_-induced hyperalgesia was prevented by treatment with a non-selective inhibitor of NOS (L-NMMA, 10 mg/kg, s.c., 30 min before the U50488 injection) **(B) **Mechanical hyperalgesia in rats was induced by an ipl. injection of PGE_2 _(100 ng/paw)_. _The antinociceptive effect of U50488 treatment at a locally effective dose (10 μg/paw, 2 h after PGE_2 _injection) after PGE_2_-induced hyperalgesia was prevented by treatment with a selective inhibitor of nNOS (N-propyl-L-arginine, 30 μg/paw, 30 min before U50488 injection). Data are expressed as the mean ± S.E.M. of 5-6 animals per group. * Indicates statistical significance compared to the vehicle group. # Indicates statistical significance compared to the U50488 treated group. *P *< 0.05, one-way ANOVA, followed by the Bonferroni correction.

The participation of NOS/NO in the peripheral antinociception produced by KOR activation was analyzed. The treatment of mice with L-NMMA (10 mg/kg, s.c, 30 min before U50488) inhibited the peripheral antinociceptive effect of U50488 (Figure 2A). Moreover, the intraplantar (ipl.) treatment of rats with n-propyl-L-arginine (30 μg/paw) also inhibited the peripheral antinociceptive effect of U50488 (Figure 2B).

### Requirement of PI3Kγ for the peripheral antinociceptive effect of KOR agonist

**Figure 3:**
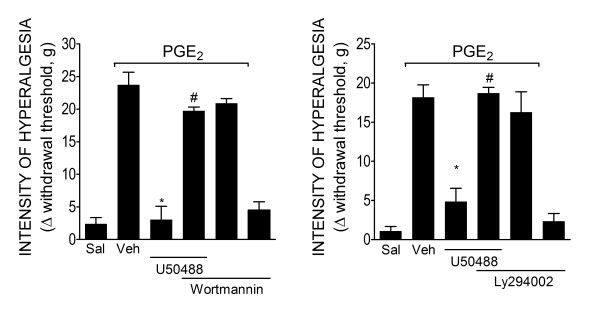
**PI3Ks mediates the peripheral antinociceptive action of KOR agonists**. (**A-B**) Mechanical hyperalgesia in rats was induced by an ipl. injection of PGE_2 _(100 ng/paw)_. _The antinociceptive effect of U50488 (10 μg/paw, 2 h after PGE_2 _injection) after PGE_2_-induced hyperalgesia was prevented by a pretreatment (30 min) with non-selective inhibitors of PI3Ks: wortmannin (3 μg/paw) or LY294002 (10 μg/paw). Data are expressed as the mean ± S.E.M. of 5-6 animals per group. * Indicates statistical significance compared to the vehicle group. # Indicates statistical significance compared to the U50488 treated group. *P *< 0.05, one-way ANOVA, followed by the Bonferroni correction.

**Figure 4:**
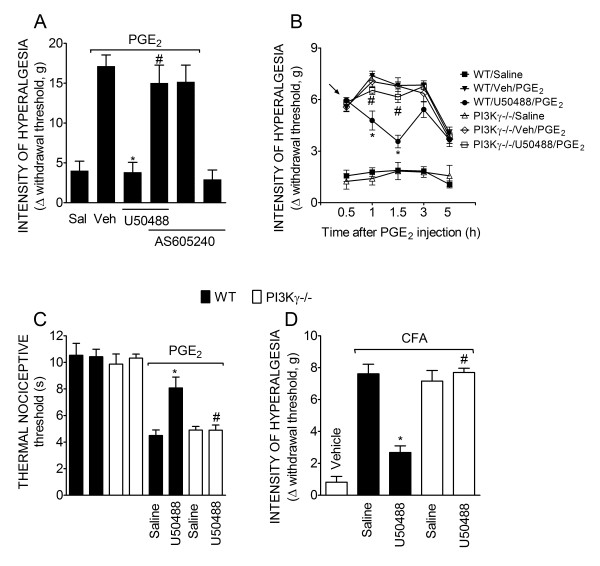
**PI3Kγ mediates the peripheral antinociceptive action of KOR agonists**. (**A**) Mechanical hyperalgesia in rats was induced by an ipl. injection of PGE_2 _(100 ng/paw)_. _The antinociceptive effect of U50488 (10 μg/paw, 2 h after PGE_2 _injection) after PGE_2_-induced hyperalgesia was prevented by treatment with a selective inhibitor of PI3K**γ **(30 μg/paw, 30 min before the U50488 injection). **(B) **Mechanical hyperalgesia was induced by the injection of PGE_2 _(30 ng/paw)_. _After 30 min, U50488 (3 μg/paw) or saline (25 μl) (indicated by the arrow) was injected in wild type or PI3Kγ-/- mice. (**C**) Thermal hyperalgesia was induced in mice by the injection of PGE_2 _(300 ng/paw). After 15 min, U50488 (10 μg/paw) was injected in the ipsilateral paws of wild type (WT) or PI3Kγ-/- mice (n = 6). Thermal hyperalgesia was evaluated 1 h after PGE_2 _injection. (D) Mice received an intraplantar injection of CFA (10 μl/paw). After 4 h, U50488 (3 μg/paw) or saline (Sal) was injected in the wild type or PI3Kγ-/- mice. Data are expressed as the mean ± S.E.M. of 5-6 animals per group. * Indicates statistical significance compared to the vehicle group. # Indicates statistical significance compared to the U50488 treated group or wild type group. *P *< 0.05, one-way ANOVA, followed by the Bonferroni correction.

To investigate if the PI3Kγ/AKT mediates the directly blockage of ongoing hyperalgesia by peripheral activation of KORs, we initially tested two non-selective inhibitors of PI3K (wortmannin and LY294002) on the peripheral antinociceptive effect of U50488. The ipl. treatment of rats with wortmannin (3 μg/paw) or LY294002 (10 μg/paw) 30 min before the U50488 (10 μg/paw) injection inhibited the U50488 peripheral antinociceptive effect (Figure 3A and 3B). Next, the effects of a selective inhibitor of PI3Kγ were tested. The treatment of rats with a selective inhibitor of PI3Kγ (AS605240, 30 μg/paw, 30 min before U50488 administration) also inhibited the antinociceptive effect of U50488 (Figure 4A). Doses of PI3Ks inhibitors were based on dose-response curves performed in a previous study [4].

The involvement of PI3Kγ in the antinociception produced by peripheral activation of KORs was further analyzed in PI3Kγ deficient mice. In agreement with the data obtained with PI3K inhibitors, U50488 did not show peripheral antinociceptive effects in PI3Kγ null mice after PGE_2_-induced mechanical and thermal hyperalgesia (Figure 4B and 4C). The PI3Kγ dependent mechanism could be extended to a more natural model of inflammatory pain since U50488 did not present peripheral antinociceptive effect in PI3Kγ null mice subjected to CFA-induced hyperalgesia (Figure 4D).

**Figure 5:**
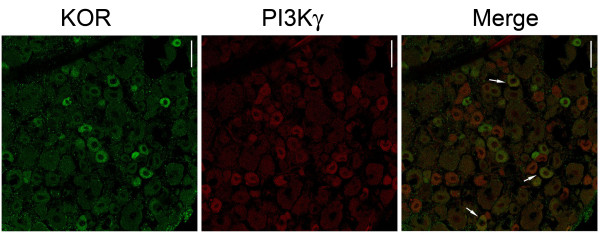
**PI3Kγ is expressed in KOR-immunoreactive neurons**. Confocal images of anti-PI3Kγ (red, left column) immunoreactivity in a population of DRG neurons from rats labeled with antibodies to KORs (green, middle column). Arrows indicate double-labeled neurons (yellow, right column). Scale bars: 50 μm.

Further supporting these results, immunofluorescence analyses of rat DRG neurons reveal that KOR-expressing neurons also express PI3Kγ (≅43%) (Figure 5). These results indicate that PI3Kγ mediates the directly blocked ongoing hyperalgesia produced by peripheral activation of KORs.

### AKT, a PI3Kγ downstream signaling kinase, is involved in the peripheral antinociceptive effect of KOR agonist

**Figure 6:**
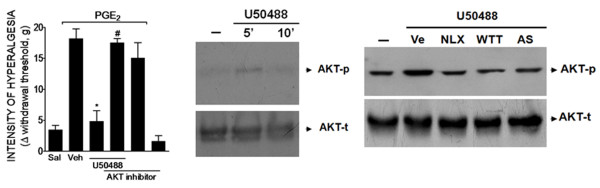
**AKT mediates the peripheral antinociceptive action of a KOR agonist**. (**A**) Mechanical hyperalgesia in rats was induced by an ipl. injection of PGE_2 _(100 ng/paw). The antinociceptive effect of U50488 (10 μg/paw, 2 h after PGE_2 _injection) after PGE_2_-induced hyperalgesia was prevented by treatment of the rat paw with a selective inhibitor of AKT (AKT inhibitor IV- 10 μg/paw, 30 min before U50488 injection). Data are expressed as the mean ± S.E.M. of 5-6 animals per group. * Indicates statistical significance compared to the vehicle group. # Indicates statistical significance compared to the U50488 treated group. *P *< 0.05, one-way ANOVA, followed by the Bonferroni correction. * *P *< 0.05, compared with vehicle treatment. (**B**) In vitro stimulation of DRG primary culture neurons from rats with U50488 (100 nM) increased the phosphorylation of AKT analyzed by Western blot. (**C**) Pre-incubation (10 min) with vehicle, wortmannin (WTT - 100 nM), AS605240 (100 nM) or naloxone (NLX- 1 μM) reduced U50488-induced AKT phosphorylation.

AKT is the most important downstream signaling kinase involved in the cellular effects of PI3Kγ [14]. Therefore, we investigated whether AKT was involved in the peripheral analgesic effect of KOR activation. Local pretreatment of rat hind paws with a selective AKT inhibitor (AKT inhibitor IV; 10 μg/paw, 30 min before U50488) reversed the peripheral antinociceptive effect of U50488 (Figure 6A). The AKT inhibitor did not affect the nociceptive threshold when injected alone in the rat paw (Figure 6A, last bar). To reinforce this in vivo observation, we analyzed the effect of U50488 on the activation of the PI3Kγ/AKT signaling pathway in cultures of dorsal root ganglion (DRGs) neurons derived from rats. The incubation of DRG cultured neurons with U50488 (100 nM) induced a rapid activation of AKT (5 min), seen as an increase in its phosphorylated form (Figure 6B). The effect of U50488 returned to baseline 10 min after incubation (Figure 6B).

Furthermore, the treatment of neurons with naloxone (NLX- 1 μM), wortmannin (100 nM) or AS605240 (100 nM) inhibited U50488-induced activation of AKT in DRG cultured neurons, suggesting the involvement of opioid receptors and PI3Kγ in the effect of U50488 (Figure 6C). Together, these results suggested that the peripheral antinociceptive effect of KOR agonists depends on the PI3Kγ/AKT that in turn might be responsible for the stimulation of nNOS/NO signaling pathway.

## Discussion

Controlling inflammatory pain by peripherally restricted opioids has been suggested as a possible approach in the development of new analgesics with fewer undesirable side effects. In the present study, we expand on this idea by showing that peripheral activation of KORs by a selective agonist directly blocks ongoing inflammatory hyperalgesia induced by PGE_2 _in rats and mice, avoiding central side effects. We also provided pharmacological and biochemical evidence that peripheral activation of KORs produced antinociception by a mechanism involving the PI3Kγ/AKT/nNOS/NO signaling pathway in primary nociceptive neurons.

Several studies have shown a peripheral antinociceptive activity of opioids during inflammatory pain. In fact, peripheral administration of MOR, KOR, and DOR (μ, κ and δ opioid receptor) selective agonists reduced mechanical or thermal hyperalgesia in different models of inflammation [2,15-17]. In this context and regarding the KOR, selective agonists with limited access to the central nervous system had been developed. They inhibited inflammatory nociception in the following models: complete adjuvant of Freund-, carrageenin- and zymosan-induced paw hyperalgesia, cancer pain, acetic acid-induced abdominal constriction in mice and formalin-induced nociception [18-20]. Interestingly, the analgesic effect of electroacupuncture seems to be mediated, at least in part, by peripheral activation of KORs [21]. Moreover, a peripherally restricted KOR agonist has completed Phase I and II clinical trials. The pharmaceutical company announced positive data with significant pain relief compared to placebo, and it was also safe and well-tolerated (unpublished observation). Despite this evidence, the mechanisms controlling antinociception produced by peripheral KOR activation are not completely understood.

Inflammatory hyperalgesia depends mainly on the sensitization (increase in excitability) of primary nociceptive neurons produced by inflammatory mediators. Therefore, peripheral KOR activation might act by reducing neuronal excitability. An important question to consider is if this effect is dependent on the KORs present on primary nociceptive neurons. The following evidence supports the hypothesis that peripheral analgesia produced by KOR activation depends on a direct effect on nociceptive neurons: a) KORs are constitutively expressed in primary nociceptive neurons as shown previously and in the present study [22]; b) activation of peripheral KORs directly blocks ongoing hyperalgesia produced by PGE_2. _The literature presents a convincing argument that PGE_2 _promotes hyperalgesia by causing a direct sensitization of primary nociceptive neurons. Therefore because U50488 inhibits the action of PGE_2_, it is likely that it acts directly on primary nociceptive neurons. Peripheral analgesics that act by inhibition of cyclooxygenase (e.g., NSAIDs) or inhibition of cytokine production (e.g., glucocorticoids) are not able to affect PGE_2_-induced hyperalgesia [23]. On the other hand, some drugs, like morphine, adenosine analogues and cannabinoids, can inhibit the ongoing sensitization induced by PGE_2 _by acting directly on peripheral nociceptive neurons [2,24,25]; c) the peripheral antinociceptive effect of morphine or KOR agonists is completely lost in capsaicin-depleted nociceptors [26,27]; and d) interestingly, it was recently shown that antinociception produced by systemic administrated opioids, at least by DOR agonists, is reduced in mice with a conditional deletion of the DOR in primary nociceptive neurons [28].

The mechanisms operating peripheral antinociceptive action of KOR agonists has been evaluated. In fact, it was shown that bremazocine, considered a selective KOR agonist, produced peripheral antinociception that is reversed by treatment with a non-selective inhibitor of NOS, suggesting the participation of NO signaling pathway [6]. Herein, we extended this previously findings showing that nNOS is most likely the NOS isoform involved in this effect. This conclusion is supported by the observation that the peripheral antinociceptive activity of U5088 was prevented by selective and non-selective inhibitors of nNOS. In this regard, the evidence is similar to that of morphine and MOR selective agonists [4]. Indeed, morphine and DAMGO (a MOR selective agonist) lost their peripheral antinociceptive effects in nNOS null mice [4,29,30]. Accordingly, the peripheral antinociceptive action of *Crotalus durissus terrificus *snake venom, which depends on KORs and DORs, is also mediated by the nNOS/NO signaling [31].

For almost twenty years the mechanism in which NO signaling is activated by peripheral opioids was unsolved. However, we recently demonstrated that the activation of this pathway is preceded by PI3Kγ/AKT signaling [4]. In fact, a morphine-induced increase in NO production by DRG neurons was blocked by incubating cells with selective inhibitors of PI3Kγ and AKT. Furthermore, morphine's peripheral antinociceptive effect was inhibited by PI3Kγ/AKT selective inhibitors, and it is not observed in PI3Kγ null mice [4]. Apart from peripheral effects, antinociception produced by systemically or centrally administered opioids was mediated, at least in part, through the stimulation of the PI3Kγ-dependent signaling pathway [32-34]. In the present, study we are showing that the peripheral antinociceptive effect of KOR is also dependent on activation of PI3Kγ/AKT signaling. Based on our previous study [4], it is likely that PI3Kγ/AKT is the link between the activation of KOR and the stimulation of nNOS/NO signaling pathway.

Although we did not investigate how AKT may modulate nNOS activity in our system, there is evidence that phosphorylation of nNOS at S1416 by AKT increases its activity [35-37]. Further studies are necessary to explore this subject in nociceptive neurons.

Another interesting question is how the NO pathway triggered by KOR activation contributes to peripheral analgesia. The peripheral analgesic activity of NO seems to be dependent on cGMP/protein kinase G and, as a last resort, from the modulation of the currents of ATP-sensitive potassium channels (KATP) [4,38]. For instance, the peripheral antinociceptive activities of opioids, NO donors and cGMP are inhibited by KATP blockers [39,40]. Additionally, in vitro morphine up-regulates KATP currents in capsaicin-sensitive neurons through the stimulation of the PI3Kγ/AKT/NO pathway [4]. Up-regulation of KATP in primary nociceptive neurons by opioids or even by cGMP seems to be associated with an alteration in the resting membrane potential, leading to hyperpolarization of these cells [4,41]. Therefore, it is likely that activation of KORs might cause a hyperpolarization of nociceptive neurons, decreasing neuronal excitability and accounting for peripheral antinociception. This hypothesis is supported by the finding that application of KOR agonists at the inflammatory site reduced spontaneous firing in polymodal nociceptors of ultraviolet-treated skin [42].

## Conclusion

In summary, our results show that peripheral activation of the KOR receptors directly blocks ongoing hyperalgesia induced by PGE_2 _in mice and rats. This effect seems to be dependent upon the activation of the KOR receptors expressed by primary nociceptive neurons, triggering the PI3Kγ/AKT which is probably responsible for the stimulation of nNOS signaling pathway. The development of peripherally restricted KOR agonists might represent a novel strategy for the treatment of inflammatory pain.

## Abbreviations

PGE_2_: Prostaglandin E_2_; (PI3Kγ): phosphoinositide 3-kinase gamma; KOR: κ-opioid receptor; MOR: μ-opioid receptor; DOR: δ-opioid receptor; NO: Nitric oxide; (nNOS): neuronal nitric oxide synthase; WT: wild type; KATP: deficient mice: -/-: ATP-sensitive potassium channels; CFA: Complete Freund's Adjuvant.

## Competing interests

The authors declare that they have no competing interests.

## Authors' contributions

TMC, ACD, GRS, CML and WAV performed the experiments. TMC and MIF participated in the design of the study. TMC, FQC and SHF conceived the study and participated in its design and coordination. TMC and SHF wrote the paper. All authors read and approved the final manuscript.
